# Super-resolution microscopy of the synaptic active zone

**DOI:** 10.3389/fncel.2015.00007

**Published:** 2015-01-30

**Authors:** Nadine Ehmann, Markus Sauer, Robert J. Kittel

**Affiliations:** ^1^Department of Neurophysiology, Institute of Physiology, University of WürzburgWürzburg, Germany; ^2^Department of Biotechnology and Biophysics, University of WürzburgWürzburg, Germany

**Keywords:** active zone, super-resolution microscopy, excitation-secretion coupling, structure-function relationships, Ca^2+^ channels

## Abstract

Brain function relies on accurate information transfer at chemical synapses. At the presynaptic active zone (AZ) a variety of specialized proteins are assembled to complex architectures, which set the basis for speed, precision and plasticity of synaptic transmission. Calcium channels are pivotal for the initiation of excitation-secretion coupling and, correspondingly, capture a central position at the AZ. Combining quantitative functional studies with modeling approaches has provided predictions of channel properties, numbers and even positions on the nanometer scale. However, elucidating the nanoscopic organization of the surrounding protein network requires direct ultrastructural access. Without this information, knowledge of molecular synaptic structure-function relationships remains incomplete. Recently, super-resolution microscopy (SRM) techniques have begun to enter the neurosciences. These approaches combine high spatial resolution with the molecular specificity of fluorescence microscopy. Here, we discuss how SRM can be used to obtain information on the organization of AZ proteins.

## Introduction

At chemical synapses, neurotransmitter release takes place at presynaptic active zones (AZs). Morphologically, AZs can be identified via their electron-dense cytomatrix—an intricate network of specialized proteins precisely organized to execute and modulate exocytosis (Zhai and Bellen, 2004; Jahn and Fasshauer, 2012; Südhof, 2012). Structure and function of AZs display varying degrees of diversity between different neuron types, between synapses of the same neuron innervating different follower cells and even between individual synapses formed by the same partner cells (Rozov et al., 2001; Atwood and Karunanithi, 2002; Zhai and Bellen, 2004; Peled and Isacoff, 2011; Ehmann et al., 2014; Paul et al., submitted). Moreover, the functional properties and the molecular composition of AZs are dynamic and can be modified in an activity-dependent manner (e.g., Wojtowicz et al., 1994; Castillo et al., 2002; Matz et al., 2010; Weyhersmüller et al., 2011). Moving from correlation to causality to clarify how different molecular architectures of AZs give rise to specific physiological properties remains a major challenge.

As an AZ contains a multitude of densely packed proteins in a small sub-cellular compartment (around 200–400 nm diameter at a central synapse; Siksou et al., 2007) diffraction-limited light microscopy delivers only very coarse structural information. Hence, morphological investigations of the fine structure and the molecular organization of AZs have mainly been restricted to electron microscopy (EM). Recently, the development of super-resolution microscopy (SRM) techniques has provided means to bypass the diffraction barrier of ~300 nm in lateral dimensions (Abbe, 1873) and to bridge the gap between conventional light microscopy and EM (for detailed recent overviews, see Hell, 2009; Patterson et al., 2010; Schermelleh et al., 2010; Galbraith and Galbraith, 2011; Sauer, 2013). These emerging technologies offer promising new options for studying nanoscopic sub-cellular structures.

Recent work has reviewed the molecular composition of AZs (Owald and Sigrist, 2009; Jahn and Fasshauer, 2012; Südhof, 2012). This perspective will focus on excitation-secretion coupling, i.e., the transduction of an electrical signal into Calcium (Ca^2+^)-dependent neurotransmitter release (Schneggenburger and Neher, 2005; Wojcik and Brose, 2007). We will summarize current information on functional determinants of the AZ and explore how the search for structural correlates can be supported by SRM to improve our mechanistic understanding of neurotransmission.

## Microscopy

Fluorescence microscopy is the method of choice for visualizing biomolecules in fixed and living cells as it enables their selective and specific detection with a high signal-to-background ratio (Lichtman and Conchello, 2005). However, while light micro-scopy is ideally suited to investigate macroscopic arrangements, it fails to uncover organizational principles at the molecular scale due to its limited spatial resolution.

EM provides substantially increased resolution, though its application is restricted to lifeless, fixed and embedded biological samples. EM studies have been instrumental in recognizing the large morphological diversity of AZs (Zhai and Bellen, 2004) and in identifying repetitive structural elements within individual, chemically fixed AZs (Pfenninger et al., 1972; Phillips et al., 2001). Moreover, alternative tissue preparation and fixation techniques have enabled analyses of filamentous AZ structures and their associated synaptic vesicles in various organisms (Landis et al., 1988; Siksou et al., 2007; Jiao et al., 2010; Wichmann and Sigrist, 2010; Fernández-Busnadiego et al., 2013). The resolving power of EM is exemplified by a classical tomographic study at the frog neuromuscular junction (NMJ). The results revealed an intricate fine structure of the AZ, which establishes a regular and precisely organized arrangement of synaptic vesicles relative to Ca^2+^ channels at release sites (Harlow et al., 2001). As more substructural details are uncovered (Szule et al., 2012), knowledge of the underlying protein species becomes increasingly desirable. Immunogold labeling provides a means to locate specific proteins in electron micrographs with nanometer resolution and has been used to examine the topology of AZs (e.g., Limbach et al., 2011). However, specific labeling with antibody-coupled gold particles is inefficient and a compromise must be made between optimal tissue preservation and structural resolution. Consequently, the ideal microscope should combine the minimal invasiveness and efficient specific labeling possibilities of optical microscopy with the high spatial resolution of EM. Technologies that merge these features, at least to a certain extent, are collectively termed SRM. These include structured illumination microscopy (SIM), stimulated emission depletion (STED) and single-molecule based localization microscopy methods, such as photo-activated localization microscopy (PALM) and *direct* stochastic optical reconstruction microscopy (*d*STORM). The techniques can be subdivided based on their principle of bypassing the diffraction barrier: deterministic approaches, such as STED, use a phase mask to define the coordinates of fluorescence emission predefined in space by the zero-node, whereas PALM and *d*STORM use stochastic activation of individual fluorophores and precise position determination (localization).

SIM relies on patterned illumination of the specimen with a high spatial frequency in various orientations providing a lateral resolution of approximately 100 nm (Heintzmann and Cremer, 1999; Gustafsson, 2000). Fortunately, SIM does not depend on any specific fluorophore properties, such as high photostability or particular transitions between orthogonal states, and can therefore be generally applied. A further modification of SIM, known as SSIM (saturated-SIM) exhibits higher spatial resolution but requires photostable samples (Gustafsson, 2005). As SIM enables multicolor 3D-imaging with standard fluorescent dyes, it has attracted considerable interest among biologists (Maglione and Sigrist, 2013).

In STED microscopy, the lateral resolution is improved by decreasing the size of the excitation point-spread-function (PSF) by stimulated emission of fluorophores at the rim of the PSF (Hell and Wichmann, 1994). Since the resolution enhancement in STED microscopy scales with the intensity of the depletion beam (Hell, 2007), only very photostable fluorophores allow spatial resolutions in the 30–50 nm range (Hell, 2007; Meyer et al., 2008). Nevertheless, STED has also been used for live-cell super-resolution imaging albeit at lower resolution (Nägerl et al., 2008; Tønnesen et al., 2014).

Single-molecule based localization microscopy techniques such as PALM, STORM and *d*STORM rely on stochastic photoactivation, photoconversion, or photoswitching of fluorophores, such that only a small subset emits photons at any given time. By fitting a 2D Gaussian function to the PSF of individual, spatially isolated emitters, their positions can be precisely localized and used to reconstruct a super-resolved image, as long as all fluorophores determining the structure of interest have been detected and localized at least once during acquisition (Betzig et al., 2006; Hess et al., 2006; Rust et al., 2006; Heilemann et al., 2008). Localization microscopy methods differ in their use of fluorescent probes: PALM is conducted with genetically expressed photoactivatable fluorescent proteins (Betzig et al., 2006; Hess et al., 2006), STORM requires photoswitchable dye pairs (Rust et al., 2006) and *d*STORM takes advantage of the reversible photoswitching of standard organic fluorophores in thiol-containing aqueous buffer (Heilemann et al., 2008; van de Linde et al., 2011). Since localization microscopy exhibits explicit single-molecule sensitivity, all approaches can deliver quantitative information on molecular distributions and even have the potential to report absolute numbers of proteins present in sub-cellular compartments (Sauer, 2013). These features provide insight into biological systems at a molecular level and can yield direct experimental feedback for modeling the complexity of biological interactions.

## Functional parameters of the AZ

Derived from the quantal hypothesis (Del Castillo and Katz, 1954) it is understood that synaptic strength, i.e., the amplitude of an excitatory postsynaptic current (EPSC), can be described by the product of three basic parameters: *N*, the number of fusion competent synaptic vesicles also termed readily-releasable vesicles (RRVs), *p*, their probability of exocytosis and *q*, usually taken to reflect postsynaptic sensitivity (Equation 1). This conceptual framework plays an important role in explaining synaptic function and plasticity (Zucker and Regehr, 2002), and identifies *N* and *p* as major functional determinants of the presynapse.

(1) EPSC=Npq

The parameter *N* can be estimated by electrophysiological means, such as high-frequency electrical stimulation or fluctuation analysis of synaptic responses (Clements and Silver, 2000). Results obtained by either approach must, however, be interpreted carefully, as additional factors complicate the analysis (Sakaba et al., 2002; Hallermann et al., 2010a). For example, asynchronous release, the kinetics of vesicle pool refilling (Hosoi et al., 2007) and postsynaptic contributions, such as receptor desensitization and saturation (Scheuss et al., 2002), can influence approximations of *N*. Hypertonic sucrose stimulation can be used as another technique to approximate *N* (Fatt and Katz, 1952; Rosenmund and Stevens, 1996). However, being independent of Ca^2+^-triggered fusion, it remains uncertain whether hypertonically released vesicles are generally also readily released under physiological conditions (Moulder and Mennerick, 2005).

Alternatively, *N* can be defined as the number of release sites, in which case *p* denotes the probability that a vesicle will fuse at a given release site (Schneggenburger et al., 2002). Nerve terminals vary greatly in size and correspondingly contain between one (e.g., at certain cortical synapses; Xu-Friedman et al., 2001) and many hundreds of AZs (e.g., at the Calyx of Held; Sätzler et al., 2002). It is therefore helpful to view the AZ as a fundamental unit of presynaptic function (Alabi and Tsien, 2012). That said, morphology and function of AZs are highly heterogeneous (Zhai and Bellen, 2004), also varying within one and the same neuron (Atwood and Karunanithi, 2002; Peled and Isacoff, 2011; Ehmann et al., 2014). Correspondingly, functional estimates of *p* at central mammalian synapses have reported both AZs operating with uniquantal release and AZs capable of multivesicular release (Tong and Jahr, 1994; Auger et al., 1998; Silver et al., 2003). To date, this next level of AZ organization has been difficult to study as specific molecular markers or structural correlates of release sites remain uncertain.

Functional estimates of *p* can be obtained with several methods that provide relative or absolute values. These include electro-physiology-based approaches such as paired-pulse stimulation or fluctuation analysis (Clements and Silver, 2000; Sakaba et al., 2002; Zucker and Regehr, 2002) and dynamic optical readouts of exocytosis or postsynaptic activation (Branco and Staras, 2009; Zhang et al., 2009; Peled and Isacoff, 2011; Marvin et al., 2013). Since *p* is highly Ca^2+^-dependent, its value for a given synaptic vesicle will be strongly influenced by the vesicle’s position relative to voltage-gated Ca^2+^ channels at the AZ (Neher, 1998; Eggermann et al., 2012).

Ca^2+^ channels are essential components of the macro-molecular exocytosis machinery. Their opening elicits Ca^2+^ influx, which serves as the fusion trigger for nearby vesicles. Early computational and functional studies introduced the concept of “microdomains” to describe transient, local regions of high Ca^2+^ concentration (Chad and Eckert, 1984; Llinás et al., 1992). Such microdomains possess complex spatial distributions of Ca^2+^ elevation, which are controlled by Ca^2+^ diffusion, Ca^2+^ buffering and the geometric arrangement of Ca^2+^ channels in the AZ membrane (Neher, 1998). Due to their major functional significance for synaptic transmission, detailed understanding of Ca^2+^ signals and the arrangement of synaptic vesicles relative to local domains is important. Using electrophysiology, modeling, Ca^2+^ imaging and Ca^2+^ uncaging, considerable quantitative information on excitation-secretion coupling has been obtained at the Calyx of Held, a large glutamatergic synapse in the mammalian auditory brainstem (Bollmann et al., 2000; Schneggenburger and Neher, 2005; Sun et al., 2007). At calyceal terminals, electrophysiology has even delivered direct functional readouts (Stanley, 1993) and estimates of AZ Ca^2+^ channel numbers (Sheng et al., 2012). Application of synthetic Ca^2+^ chelators with different binding rates [BAPTA (1,2-bis(2-aminophenoxy)ethane-N,N,N’,N’-tetraacetic acid) and EGTA (ethylene glycol-bis(2-aminoethylether)-N,N,N’,N’-tetraacetic acid)] can differentiate between very tight (“nanodomain”, <100 nm) and larger distance (“microdomain”, >100 nm) coupling regimes of synaptic vesicles and Ca^2+^ channels (Eggermann et al., 2012). By combining data from such investigations, the vesicle-Ca^2+^ channel topography has now been modeled at several mammalian central AZs (Meinrenken et al., 2002; Schmidt et al., 2013; Vyleta and Jonas, 2014). While it would be desirable to study the ultrastructural organization underlying coupling modes directly, information on the exact arrangement of Ca^2+^ channels derived from EM is sparse (Feeney et al., 1998; Holderith et al., 2012; Indriati et al., 2013). Conventional light microscopy, in turn, cannot measure the physical distance between channels and vesicles or resolve whether the Ca^2+^ signal is shaped by a single channel (Augustine et al., 1991; Stanley, 1993) or the superposition of multiple channels (Borst and Sakmann, 1996).

There appears to be no general map of synaptic vesicle and Ca^2+^ channel arrangements at the AZ. In fact, vesicle-channel coupling may differ significantly at AZs belonging to the same neuron (Rozov et al., 2001) and at single presynaptic terminals over time (Fedchyshyn and Wang, 2005; Erazo-Fischer et al., 2007; Wong et al., 2013). Before a synaptic vesicle becomes fusion competent, the release machinery must build up a primed state (Wojcik and Brose, 2007). In addition to such “molecular priming”, evidence also suggests that “positional priming”, i.e., moving primed vesicles closer to Ca^2+^ channels, can contribute to a heterogeneous *p* of RRVs (Neher and Sakaba, 2008). However, information on spatial relationships of AZ molecules in these distinct states has not yet been collected. Importantly, proteins which influence AZ function and plasticity by tightening vesicle-Ca^2+^ channel coupling have been identified in fly and mouse (Kittel et al., 2006; Yang et al., 2010). Investigating the organization of such key components relative to other AZ constituents should help to improve our mechanistic understanding of AZ structure-function relationships.

## SRM of the AZ

Quantitative information on functional determinants of the AZ has mainly been derived from large, electrophysiologically accessible presynaptic terminals, such as the Calyx of Held (Forsythe, 1994; Meinrenken et al., 2002; Neher and Sakaba, 2008). While sophisticated electrophysiology has extended direct studies of transmitter release to smaller terminals (see e.g., Hallermann et al., 2003; Rancz et al., 2007; Bucurenciu et al., 2008), there remains an obvious demand for correlative structural information.

Here, SRM techniques can be expected to make a significant contribution. Several SRM studies, mostly conducted in cell culture, have provided indirect information on AZ function by analyzing the vesicle cycle. In one of its first biological applications, STED microscopy showed that the vesicular Ca^2+^ sensor Synaptotagmin remains clustered in isolated patches following exocytosis in cultured neurons (Willig et al., 2006). Subsequent work introduced live cell STED imaging to visualize synaptic vesicle movement between and within presynaptic boutons (Westphal et al., 2008), while multicolor imaging has been used to differentiate molecularly-defined synaptic vesicle pools at calyceal synapses in rat brain tissue (Kempf et al., 2013). Focussing on Syntaxin as a component of the vesicle fusion machinery, two independently conducted investigations using STED and *d*STORM provided detailed information on its arrangement in clusters at the plasma membrane of PC12 cells (Sieber et al., 2007; Bar-On et al., 2012). Moreover, 3-D applications of STORM and PALM have been utilized to investigate vesicle endocytosis by Clathrin nanostructures in cultured cell lines (Jones et al., 2011; Sochacki et al., 2012).

**Figure 1:**
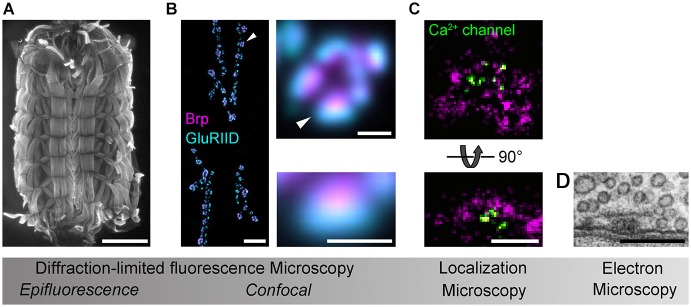
**Imaging *Drosophila* neuromuscular AZs**. Gradual increases in spatial resolution show **(A)** a *Drosophila* larval preparation imaged with epifluorescence microscopy (phalloidin staining); **(B)** a confocal image of the glutamatergic neuromuscular junction (left panel), a single bouton (upper panel) and an individual synapse (lower panel) stained against the AZ protein Brp (magenta) and the postsynaptic glutamate receptor subunit GluRIID (cyan; arrowheads indicate enlarged regions); **(C)** *d*STORM images of AZs stained against Brp (C-terminal epitope, magenta) and Ca^2+^ channels (nanobody recognizing a GFP-tagged α1-subunit, Cac^GFP^; Kawasaki et al., 2004) viewed *en face* (optical axis perpendicular to AZ membrane, upper panel) and from the side (optical axis parallel to AZ membrane, lower panel; cf. **D**); **(D)** an electron micrograph of the AZ cytomatrix and opposed pre- and postsynaptic membranes. Electron micrograph kindly provided by C. Wichmann and S.J. Sigrist. Scale bars: **(A)** 1 mm; **(B)** 10µm (NMJ), 1µm (bouton), 500 nm (synapse); **(C,D)** 200 nm.

Analysis of the AZ nanoarchitecture in tissue was first carried out with SRM by using STED at the *Drosophila* NMJ. Beginning with the identification of Bruchpilot (Brp) as a major component of the AZ cytomatrix (Kittel et al., 2006; Wagh et al., 2006), subsequent work described the polarized, elongated orientation of this large filamentous protein and resolved the organization of further AZ components, such as Ca^2+^ channels, Syd-1, Liprin-α and RIM binding protein (RBP) relative to the Brp hub (Fouquet et al., 2009; Owald et al., 2010; Liu et al., 2011). This has generated an increasingly detailed picture of the protein scaffold at *Drosophila* AZs (Maglione and Sigrist, 2013), which is currently being extended by photobleaching microscopy techniques (PiMP, photo-bleaching microscopy with nonlinear processing; Khuong et al., 2013) and SRM via *d*STORM (Figure 1; Ehmann et al., 2014, Paul et al., submitted). In a separate effort, STORM was used to measure the axial positions of the AZ-specific proteins RIM1, Piccolo and Bassoon at synapses in mouse brain tissue (Dani et al., 2010). It is of obvious interest to compare such AZ topographies from different synapses, to identify conserved and specialized principles of organization and to test whether these are causally linked to functional diversity.

Extending beyond descriptive ultrastructural studies, microscopy can contribute to identifying structural correlates of synaptic function (Wojtowicz et al., 1994). Considering their fundamental impact on neurotransmission there has thus been a long standing motivation to resolve the nanoscopic organization of Ca^2+^ channels at the AZ. However, to date little direct information has been collected on their ultrastructural distribution (Haydon et al., 1994; Feeney et al., 1998; Holderith et al., 2012; Indriati et al., 2013). Notably, a recent study at hippocampal neurons elegantly combined Ca^2+^ imaging with EM to estimate the number of Ca^2+^ channels contributing to one microdomain and to identify a close correlation between the number of docked vesicles, AZ area and *p* (Holderith et al., 2012). Combining STED with molecular manipulations and electrophysiology has identified functional roles of the AZ proteins Brp and RBP in the recruitment and spatial arrangement of Ca^2+^ channels to promote *p* at the *Drosophila* AZ (Kittel et al., 2006; Hallermann et al., 2010b; Liu et al., 2011). Moreover, dynamic reorganizations of Brp accompany rapid AZ strengthening and increase the number of release sites during homeostatic synaptic plasticity (Weyhersmüller et al., 2011). Similarly, studies at mammalian hair cell synapses have demonstrated a role of the AZ protein Bassoon, functionally related to Brp (Hallermann and Silver, 2013), in shaping Ca^2+^ channel arrangement and establishing release sites (Frank et al., 2010).

Despite the high spatial resolution provided by SRM, estimates of protein abundance are mainly obtained from fluorescence intensity measurements and therefore deliver only relative values. However, quantitative information on endogenous protein copies, in addition to their spatial organization, is required for a comprehensive mechanistic understanding of AZ structure-function relationships. While stepwise photobleaching can be used to count low protein numbers (Ulbrich and Isacoff, 2007) the densely packed protein assembly at the AZ requires alternative methods. Several recent reports have addressed this issue.

Wilhelm et al. combined quantitative biochemistry with EM and STED to estimate average protein copies and to localize these to specific sub-cellular regions of biochemically isolated presynaptic terminals (Wilhelm et al., 2014). This approach has delivered a wealth of quantitative information on presynaptic proteins. However, it does not connect structural features with functional properties at the single synapse level.

Since localization microscopy is an explicit single-molecule imaging technique, it can be used to obtain quantitative information on both the spatial distribution and the copy number of labeled proteins *in situ*, as long as antibody binding features (e.g., in *d*STORM) or fluorescent protein expression and folding properties (as e.g., in PALM) are taken into account. By engaging *d*STORM, this principle was recently utilized to study the nanoscopic arrangement of endogenous Brp proteins at AZs in tissue (Ehmann et al., 2014). The results provided an estimate of the number of Brp copies per AZ and were correlated with electrophysiological features to offer an interpretation of how the protein’s organization is linked to AZ function.

These current developments open up new perspectives for clarifying how functional properties are encoded in the protein architecture of AZs. Logical next steps could include searching for molecular determinants of vesicle release sites and quantitative ultrastructural studies of Ca^2+^ channel-vesicle topographies.

## Outlook

Despite a gradually emerging comprehensive protein catalog, we still lack basic information describing how the nanoscopic organization of proteins at the AZ gives rise to neurotransmission. Arguably, this is due to the diffraction-limited resolution of conventional light microscopy, which has hindered access to the spatial nanodomain in a physiologically relevant experimental setting.

Several SRM techniques now exist that have the capacity to localize proteins on the nanometer scale and to resolve components of macromolecular assemblies in their native environment. In this context, we believe that localization microscopy is of particular value, as it can be used to provide direct access to molecular coordinates and to count endogenous protein epitopes (Specht et al., 2013; Andreska et al., 2014; Ehmann et al., 2014). We expect that combining such quantitative information on protein organization with results from electrophysiology will contribute to a better understanding of the molecular mechanisms controlling AZ function. In addition, other correlative approaches, such as pairing SRM with biochemistry (Wilhelm et al., 2014), EM (Watanabe et al., 2011; Löschberger et al., 2014) and array tomography (Nanguneri et al., 2012) hold great promise for uncovering multiprotein architectures.

Harnessing the full potential of SRM will require expanding the repertoire of robust test samples and introducing optimized analytical tools (Bar-On et al., 2012). Likewise, small fluorescent probes with both efficient and specific binding properties will have to be developed to allow for simultaneous visualization of multiple targets in their native settings (Sauer, 2013). As already common practice in EM, users of SRM have to accept that fluorophores, labeling protocols and sample preparations need to be optimized for each new target molecule under investigation.

Dynamic, live-cell SRM remains challenging. As a rule of thumb, spatial resolution always comes at the cost of temporal resolution. Therefore, imaging complex structures, such as the cytoskeleton of a whole cell, requires several minutes acquisition time at a lateral resolution of about 20 nm. This clearly limits the obtainable dynamic information. In contrast, modified SIM can easily resolve the movement of microtubules in entire living cells, albeit at lower spatial resolution (Chen et al., 2014). Hence, future efforts will have to optimize the trade-off between imaging area, temporal information and spatial resolution in order to monitor dynamic protein re-arrangements at the AZ directly. In principle, fluorescent protein-based SRM techniques offer the possibility of *in vivo* imaging in fully intact organisms. However, the feasibility of such applications must take into account light scattering and aberration in biological tissue, less amenable photophysical properties of fluorescent proteins compared with organic fluorophores and possible physiological alterations induced by recombinant protein expression (Sauer, 2013).

Despite its capacity to resolve multiprotein structures, so far relatively few studies have engaged SRM to study synaptic AZs. We anticipate that this situation will change as SRM techniques become increasingly available and affordable (Holm et al., 2014). Progress in efficient and stoichiometric labeling of endogenous proteins, together with the development of sample preparations that accurately preserve the molecular details of interest, will further advance SRM to shed light on the AZ.

## Conflict of interest statement

The authors declare that the research was conducted in the absence of any commercial or financial relationships that could be construed as a potential conflict of interest.
